# A Defect Dataset for Electrode Coating Manufacturing

**DOI:** 10.1038/s41597-025-06419-1

**Published:** 2026-02-14

**Authors:** Vignesh Sampath, Andrew S. Lee, Samuel David Miller, Noah H. Paulson, Yuepeng Zhang, Logan Ward

**Affiliations:** 1 Data Science and Learning Division, Argonne National Laboratory, Lemont, IL USA; 2 Applied Materials Division, Argonne National Laboratory, Lemont, IL USA

**Keywords:** Research data, Energy and society

## Abstract

Electrode is a key component of many energy storage and energy conversion devices such as batteries and fuel cells. Defects in electrodes can significantly influence device performance and reliability and thus need to be monitored and eliminated during the electrode manufacturing process. Advancements in in-line metrology, computer vision, and machine learning have enabled the development of integrated hardware-software systems for automated defect detection and diagnostics. While several manufacturing domains have published defect datasets to support such efforts, publicly available datasets specific to electrode coating processes are not available. To fill this gap and support research on defect detection for automated coating processes, we present CoatingVision, a comprehensive dataset of slot-die coating images with labeled defect types. This dataset supports a diverse range of image recognition tasks, including defect segmentation, defect detection, and multi-label classification. It includes high-resolution images with associated labels for common defects such as surface cracks, delamination cracks, pinholes, and unclassified defects. To facilitate benchmarking and reproducible research, CoatingVision is packaged with an open-source codebase that enables comparative evaluation of AI models and hyperparameter configurations. The dataset has been meticulously curated to ensure high quality and consistency, providing researchers with reliable data for training and evaluating computer vision models. With over 2,200 image samples under various processing conditions, CoatingVision offers a robust foundation for developing automated defect detection systems. It promotes deeper insights into defect formation in coating manufacturing processes, which can be used to advance various coating-related applications including batteries and fuel cells.

## Background & Summary

In the fields of energy storage and advanced manufacturing, the fabrication of high-quality electrode coatings is crucial for ensuring the performance, reliability, and longevity of energy conversion and storage systems^1^. As the demand for efficient and sustainable energy solutions rises, the quality of the electrode coating process has become a key factor in achieving optimal energy storage performance^2^. Defects in the electrode coatings, whether occurring during production or application, can significantly reduce the efficiency, capacity, and lifespan of these systems^3^. Consequently, maintaining stringent quality control throughout the manufacturing process is essential to safeguard the performance and durability of the final product.

To meet the growing demand for high-performance energy storage systems, the development of automated quality assurance technologies for electrode coating has become increasingly important^4^. By integrating advanced computer vision and machine learning methods into manufacturing pipelines, real-time monitoring and precise evaluation of electrode coatings have become feasible^5^. These technologies enable the rapid detection and classification of defects, reducing reliance on manual inspection, enhancing scalability, and ensuring the production of high-quality coatings suited for large-scale industrial applications.

**Fig. 1:**
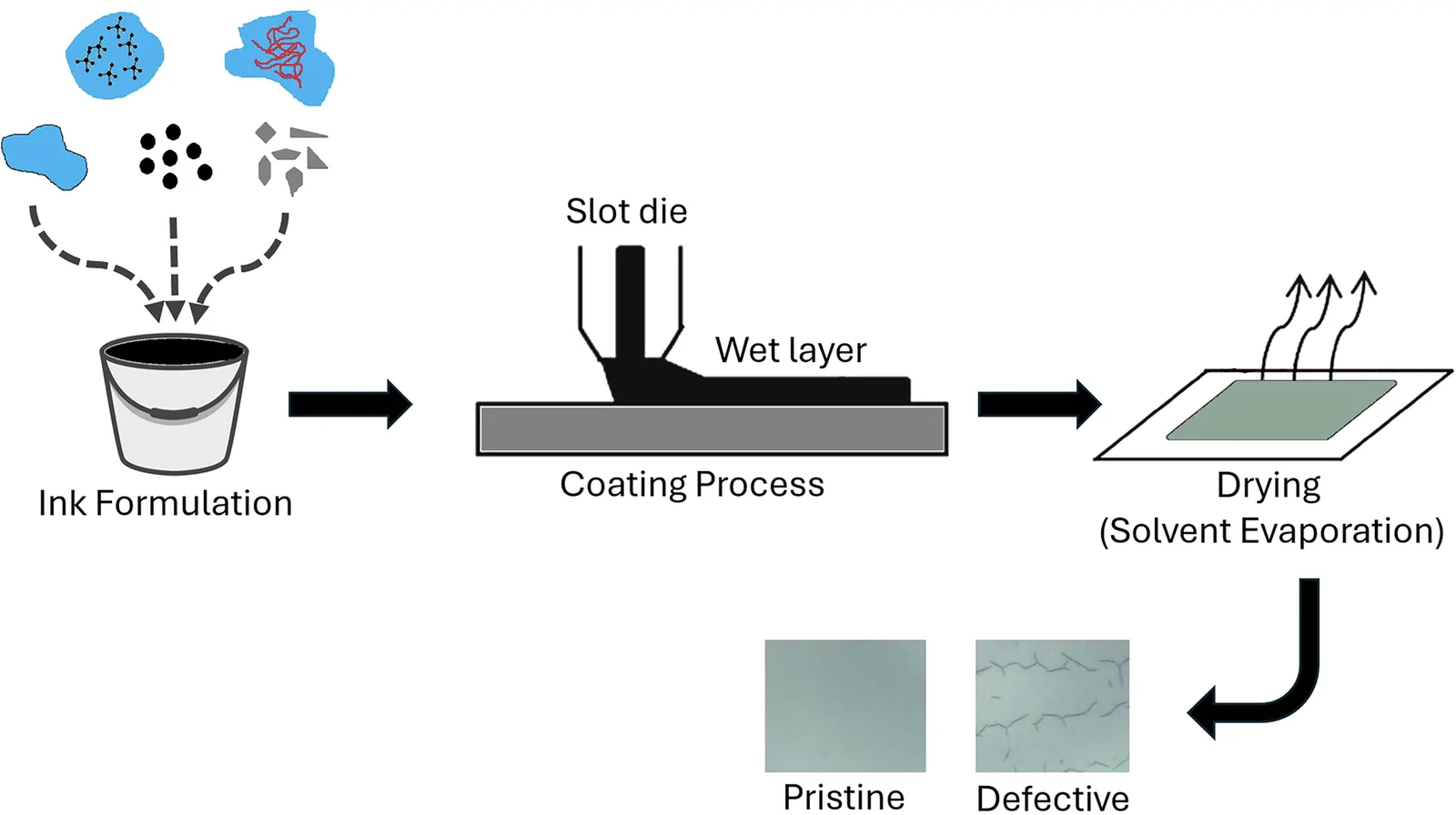
High-level schematic of the electrode coating process, highlighting slurry preparation, die slot coating, drying, and compaction.

The coating process is inherently complex, with multiple parameters influencing the final electrode quality^1^. In general, electrode manufacturing involves mixing the active material^6^, binder^7^, and conductive agent to prepare a slurry^8^, which is subsequently coated onto a substrate^9^. After drying to remove the solvent, an electrode sheet is obtained. The coating is then compacted and densified before the sheet is cut or slit to the desired dimensions (see Fig. 1). Variations in material properties, processing conditions, and environmental factors can introduce defects that degrade the performance and lifespan of energy storage devices.

Defects in electrode coatings, such as surface cracks, delaminated cracks, pinholes, and other irregularities, often arise due to the interactions between these various factors^10^. Due to the complex nature of electrode coating, understanding the fundamental mechanisms governing film formation and stability remains an active area of research. The interplay between thermodynamics and hydrodynamics plays a crucial role in governing the behavior of coating slurries during drying, compaction, and densification. However, the precise mechanisms leading to defect formation are still not fully understood, as they depend on a combination of material properties, processing conditions, and environmental factors.

Previous studies have suggested that during the drying process, solvent evaporation causes a reduction in particle–particle distances, leading to capillary pressure that drives particle cohesion. As the film continues to shrink, a porous network forms, and liquid-filled pores begin to empty, weakening cohesion between affected particles. This phenomenon results in surface cracks, which typically originate at the top layer and propagate toward the substrate^11–13^. The likelihood of cracking increases with larger particle sizes, wider particle size distributions, and thicker coatings. High drying temperatures tend to promote crack formation, whereas humidified drying conditions may help mitigate cracking^14–18^.

At the interface between the coating and the substrate, the loss of cohesion can lead to delamination cracks. Delamination occurs when the adhesion between film particles and the substrate is insufficient to counteract the internal stresses generated during drying. The tendency for delamination depends on the adhesive properties of the coating material, the roughness of the substrate, and the overall mechanical integrity of the film^19^.

From an electrochemical perspective, defects in electrode coatings can influence ion transport, electronic conductivity, and overall cell performance. While some studies^20^ suggest that minor defects such as pinholes and small agglomerates may not significantly degrade electrochemical performance, the precise correlation between defect type, size, and distribution remains an open question. Research is still ongoing to determine the optimal defect tolerance thresholds and their cumulative impact on cycle life, capacity retention, and rate capability.

By employing deep learning models, the identification and characterization of coating defects can be significantly accelerated, providing an opportunity for more efficient and consistent quality control^21–23^. Such advancements are crucial for enhancing manufacturing processes, particularly in fields like electrode production, where defects can lead to costly failures in energy storage systems. The success of these models largely depends on access to high-quality, annotated datasets that support a variety of computer vision tasks. These datasets are crucial for training models that can effectively identify and classify defects under diverse production conditions. To fill this gap, we present a comprehensive dataset designed to accelerate research in automated defect detection systems for electrode fabrication quality control. Our dataset encompasses a broad spectrum of image recognition tasks, including defect segmentation, detection, and multi-label classification. It includes high-resolution images with labels for common defects such as surface cracks, delamination cracks, pinholes, and other defects, providing a solid foundation for advancing research in both battery and fuel cell production technologies.

## Literature Review

Defect detection has been widely explored in various industrial domains, with multiple publicly available datasets facilitating advancements in computer vision-based inspection. These datasets can be broadly grouped based on their application domains, defect types, and data characteristics.

### Construction and infrastructure

Datasets in this domain primarily focus on crack detection in materials such as masonry, road pavement, concrete, and ceramic tiles. The Masonry dataset^24^ includes images of masonry walls, featuring a wide range of crack sizes, from small patches affecting a few bricks to larger-scale cracks spanning entire walls. These images were sourced from both the internet and actual masonry buildings in Groningen, Netherlands, offering a diverse collection of real-world data. In a similar vein, the CFD dataset^25^ captures urban road surface conditions in Beijing, China, with detailed, hand-labeled ground truth contours for cracks, providing high-quality data for road maintenance and inspection. Another well-established dataset, CrackTree200^26^, includes images of cracks collected from pavements and buildings, with challenges such as varying lighting conditions, shadows, occlusions, and noise that complicate the detection process. In a complementary context, the Volker^27^ and DeepCrack^28^ datasets also focus on cracks in building structures, addressing issues such as low contrast and noisy environments, which are common in real-world data and present significant challenges for automated detection systems. The Ceramic Cracks Database^29^, curated by students at the University of Pernambuco, expands the scope by including images of cracks on ceramic building facades, capturing defects across various textures and colors, further enriching the dataset diversity. The SDNET2018^30^ dataset offers images from concrete bridge decks, walls, and pavements, including cracks that range from narrow fissures as small as 0.06 mm to significantly wider gaps of 25 mm. The dataset provides real-world scenarios that include obstructions like shadows, surface roughness, scaling, and background debris, which add complexity to automated crack detection systems. Likewise, the Rissbilder dataset^27^ contains a wide range of architectural crack types, showcasing the diversity in crack patterns that may appear in different structural contexts. Crack500^31^, collected at Temple University, further adds to the dataset landscape by using smartphone sensors to capture cracks, reflecting the growing trend of utilizing consumer-grade devices for field data collection. The GAPS dataset^32^, collected under dry and warm conditions in 2015, provides images of various cracks found in urban environments, offering another valuable resource for the detection of road and pavement defects.

#### Electronics manufacturing

Expanding into other industries, the KolektorSDD^33^ dataset focuses on defective electronic converters, capturing small cracks in the plastic casing of the electronic components. With 52 defective and 347 non-defective samples, this dataset addresses a niche application in electronic defect detection. Yang *et al*.^34^ introduced a Heat Sink Surface Defect dataset, which contains 1000 surface images of gold-plated tungsten copper alloy heat sinks, specifically aimed at detecting defects in high-precision components used in electronics and cooling systems.

#### Steel and wood industry

The NEU-DET dataset^35^, released by Northeast University, includes gray-scale images depicting surface defects in hot-rolled steel strips. This dataset categorizes defects into three main types: patches, inclusions, and scratches, with 300 images for each defect, offering high-quality data for the steel industry. The Severstal dataset^36^, one of the largest industrial datasets, features over 12,000 high-resolution grayscale images of steel sheet defects, annotated with pixel-wise masks by technical experts, covering various defect classes like cracks and scratches.In the wood industry, Pavel *et al*.^37^ introduced a large-scale dataset of sawn timber surface defects, containing more than 43,000 labeled images of surface defects across 10 different types, including splits, knots, and holes. This extensive dataset is pivotal for quality control in the timber industry, where surface integrity is crucial for determining material usability in manufacturing and construction. The concise overview of the various datasets used for defect detection across multiple industries is presented in Table 1.

**Table 1:** Summary of Open-Source Defect Detection Datasets Across Industries.

Dataset	Industry	Material	Size	Resolution	Tasks
NEU^35^	Steel	Steel	1800	200 × 200	Object detection, Segmentation
Severstal^36^	Steel	Steel	12568	1600 × 256	Segmentation
Pavel *et al*^37^.	Wood	Wood	4000	2800 × 1024	Object detection
Volker^27^	Construction	Concrete/stone	427	512 × 512	Segmentation
DeepCrack^28^	Construction	Concrete	443	544 × 384	Segmentation
CFD^25^	Road Pavement	Road	118	480 × 320	Segmentation
CrackTree200^26^	Road Pavement	Road	175	800 × 600	Segmentation
Masonry^24^	Construction	Concrete	240	224 × 224	Classification, Segmentation
Ceramic^29^	Construction	Ceramic tiles	167	256 × 256	Segmentation
SDNET2018^30^	Construction	Concrete	1411	4032 × 3024	Classification
Rissbilder^27^	Construction	Concrete	2736	512 × 512	Object detection, Segmentation
Crack500^31^	Road Pavement	Road	3126	2000 × 1500	Segmentation
GAPS 384^32^	Road Pavement	Road	383	640 × 540	Segmentation
KolektorSDD^33^	Electronics	Plastic casing	399	500 x ~1250	Segmentation
Heat Sink^34^	Electronics	Gold-plated tungsten-copper alloy	1000	320 × 320	Segmentation

To the best of our knowledge, no defect dataset specifically related to electrode coatings has been published. Our study introduces the first such dataset, filling the gap. By utilizing the slot die coating defect datasets, manufacturers can build robust deep learning models that enable automated defect diagnostics and autonomous decision-making. These models could optimize production parameters to reduce defects or even halt production in the presence of critical anomalies, improving production safety and efficiency, minimizing material waste, and enhancing sustainability in electrode manufacturing. These advancements lay the groundwork for smart factories, where data-driven insights help optimize every step of the production cycle, ensuring that high-performing energy storage technologies are consistently delivered.

## Methods

To create a CoatingVision dataset for defect detection in slot-die-coated films, we varied experimental parameters to generate coated samples with different quality levels. These parameters were carefully controlled to simulate real-world manufacturing conditions and help identify trends in defect formation.

### Coating process

The coating process was carried out using a Benchtop slot-die coater (VectorSC, *FOM Technologies*) with a constant coating speed of 0.5 m/min. The coatings were deposited on a polytetrafluoroethylene (PTFE) substrate under different coating gaps (i.e., the distance between die head and substrate), which varied between 600 µm and 1100 µm with a 100 µm increment. The coating width and distance is 25 mm and 75 mm, respectively. After coating, the samples were dried in air for 40 minutes, followed by an additional 4 minutes drying at 80 °C in an oven. The air-dry step was introduced to avoid extraneous defect generation during sample transfer from coater to oven. All samples were handled in a consistent manner to ensure consistency.

### Ink coating parameters

The coating inks contain Vulcan carbon particles, mixed water and isopropyl alcohol (IPA) solvents, and Nafion binders. To observe how ink parameters influence defect formation in coating and drying, we varied the solvent properties of the inks such as dielectric constant, evaporation rate, and solubility by modifying the ratio of water and IPA. This parameter, alongside the varied coating gap, contributed to the observed coating quality differences between samples. Initial trends suggest that defects increased with larger coating gaps and higher water/alcohol ratios, which helped establish the ink/coating parameters suitable for the development of experimental protocols.

### Data collection methods

**Fig. 2:**
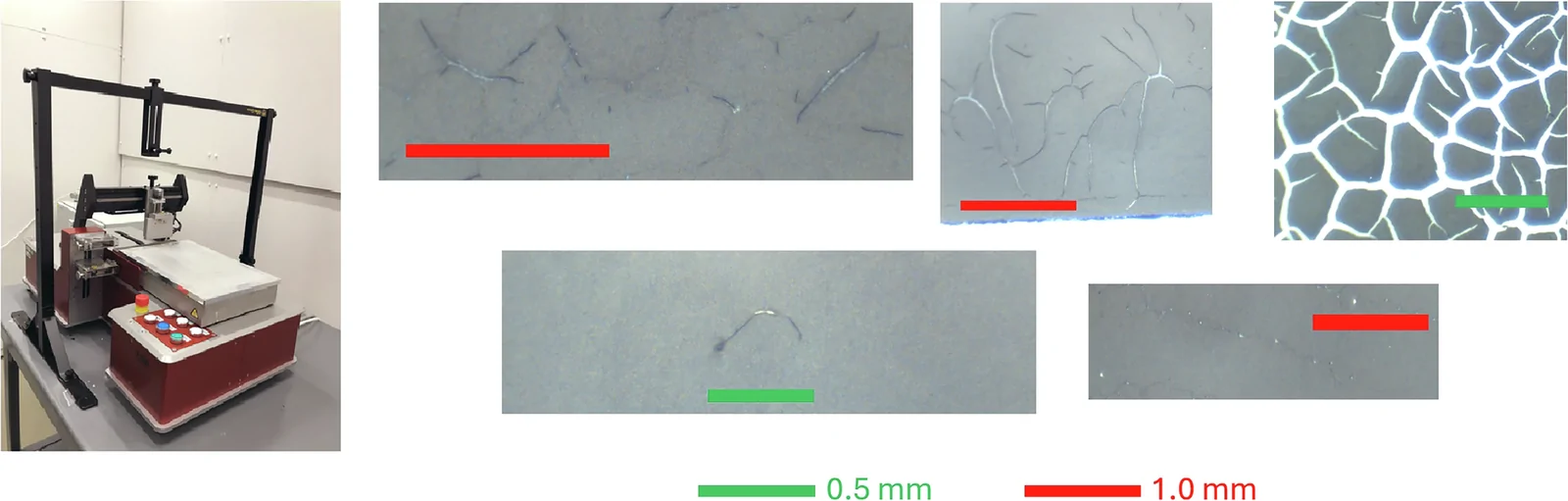
Schematic of the experimental setup and surface defect images captured using a Pixelink PL-D753CU camera with LED lighting.

Surface defects of the coating samples were captured using a Pixelink PL-D753CU area camera paired with a Navitar 12x zoom lens. The camera was positioned perpendicular to the sample surface, with the lens set to its minimum focusing distance to capture high-resolution optical images of the defects (see Fig. 2). To ensure optimal lighting and minimize shadows, LED lamps were placed on both sides of the camera. Due to the light-absorption nature of the carbon blacks in the coatings, the camera shutter speed was adjusted to allow for sufficient exposure, and the camera’s sensitivity was set to its highest setting to ensure adequately bright images.

### Data annotation methods

**Fig. 3:**
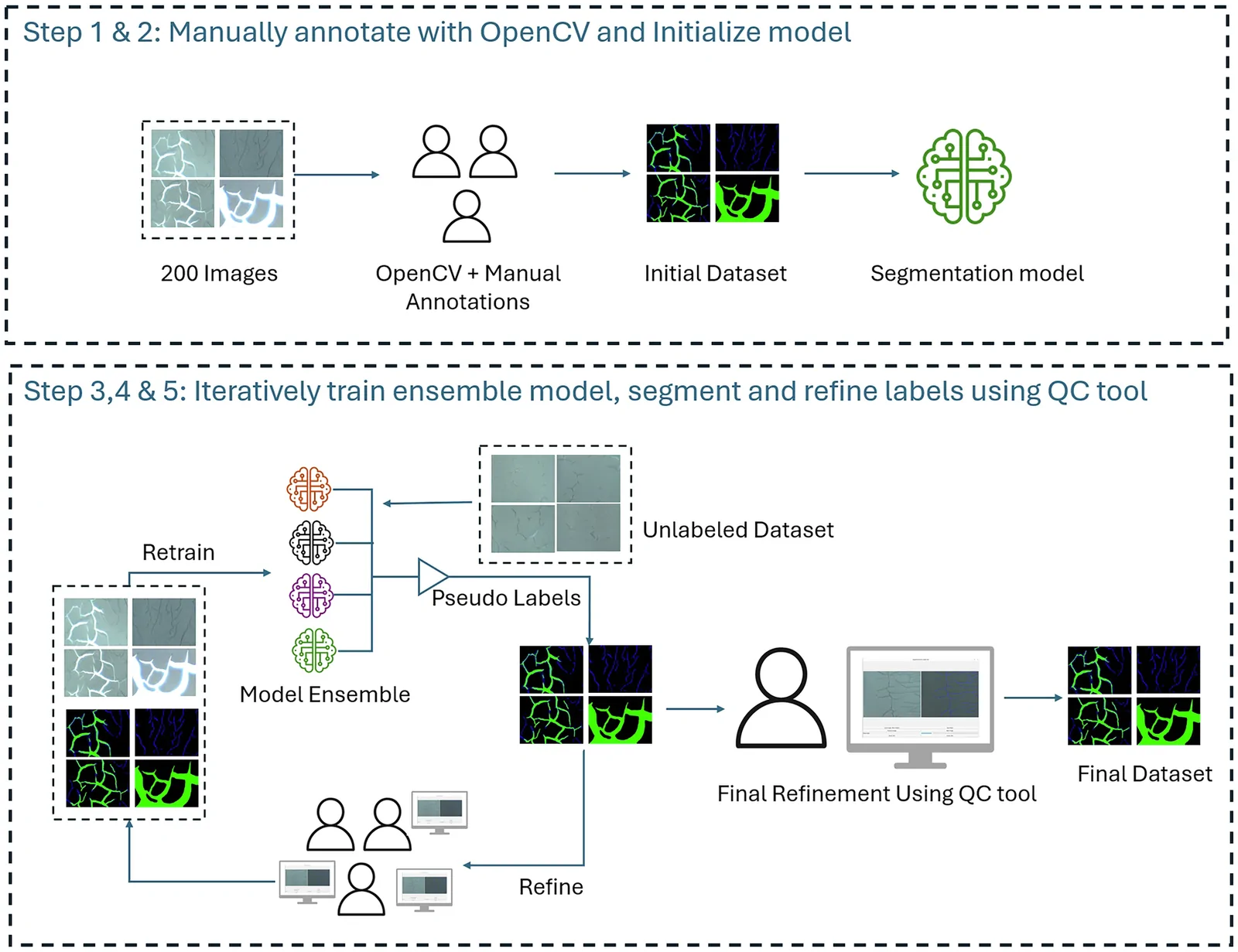
Workflow of the iterative annotation process for the CoatingVision dataset.

The annotation process for the CoatingVision dataset follows an iterative pipeline aimed at continuously improving the quality and accuracy of the segmentation models (see Fig. 3). This process is designed to generate high-quality labeled data for the development and evaluation of automated defect detection systems in electrode coatings. Below is the detailed workflow:

### Motion blur simulation and image extraction

To emulate the real-time production process and capture the effect of motion during the coating process, the data was recorded as video with a degree of motion blur. This motion blur simulated the dynamic nature of the manufacturing environment, which is often characterized by high-speed motion. From the recorded video footage, individual frames were extracted, which were then converted into high-resolution images for analysis. This allowed the model to analyze data that mirrors the conditions found in actual production environments, where motion blur is often present due to the high-speed coating process.

### Duplicate removal and hashmap filtering

To ensure the uniqueness of the dataset and avoid redundant data, a hashmap-based filtering method was applied. This technique helped identify and remove duplicate images from the extracted frames, ensuring that each image in the dataset represented a distinct instance, free from repetition. By utilizing this filtering method, the dataset quality was improved, and potential biases from duplicate data were eliminated, ensuring more accurate training and validation of segmentation models.

#### Step 1: Preprocessing and initial feature extraction

The raw images collected from the slot-die-coated electrode samples are preprocessed using traditional OpenCV-based techniques. These include morphological operations, thresholding, and contour analysis. These preprocessing steps are applied to enhance and extract key features from the images, such as surface cracks, delamination cracks, pinholes, and other miscellaneous defects. This step ensures that the images are adequately prepared for the segmentation task, highlighting relevant structures while eliminating unnecessary background noise.

#### Step 2: Initial segmentation model training

With the preprocessed dataset, three different segmentation models are trained to learn to distinguish between defective and non-defective regions in the images. These models are trained using a combination of pixel-wise labeling and multi-label classification techniques to identify and segment various types of defects, including cracks, delamination, pinholes, and other defects common in slot-die coatings. The output of each model is a set of segmentation masks that highlight the predicted regions of interest, which include both the location and type of defects.

#### Step 3: Generation of pseudo-labels

After training, the three segmentation models (U-Net,FPN and Linknet) are used to infer defect locations in new, unannotated data. The resulting segmentation masks from these models are treated as pseudo-labels. These pseudo-labels serve as the initial predictions of the segmentation models on the unannotated images, representing the models’ best attempt at identifying defects. The ensemble of these models’ predictions helps to provide a more reliable first-pass estimate for the defect locations, benefiting from the combined strengths of multiple model architectures and hyperparameters.

#### Step 4: Manual error correction

**Fig. 4:**
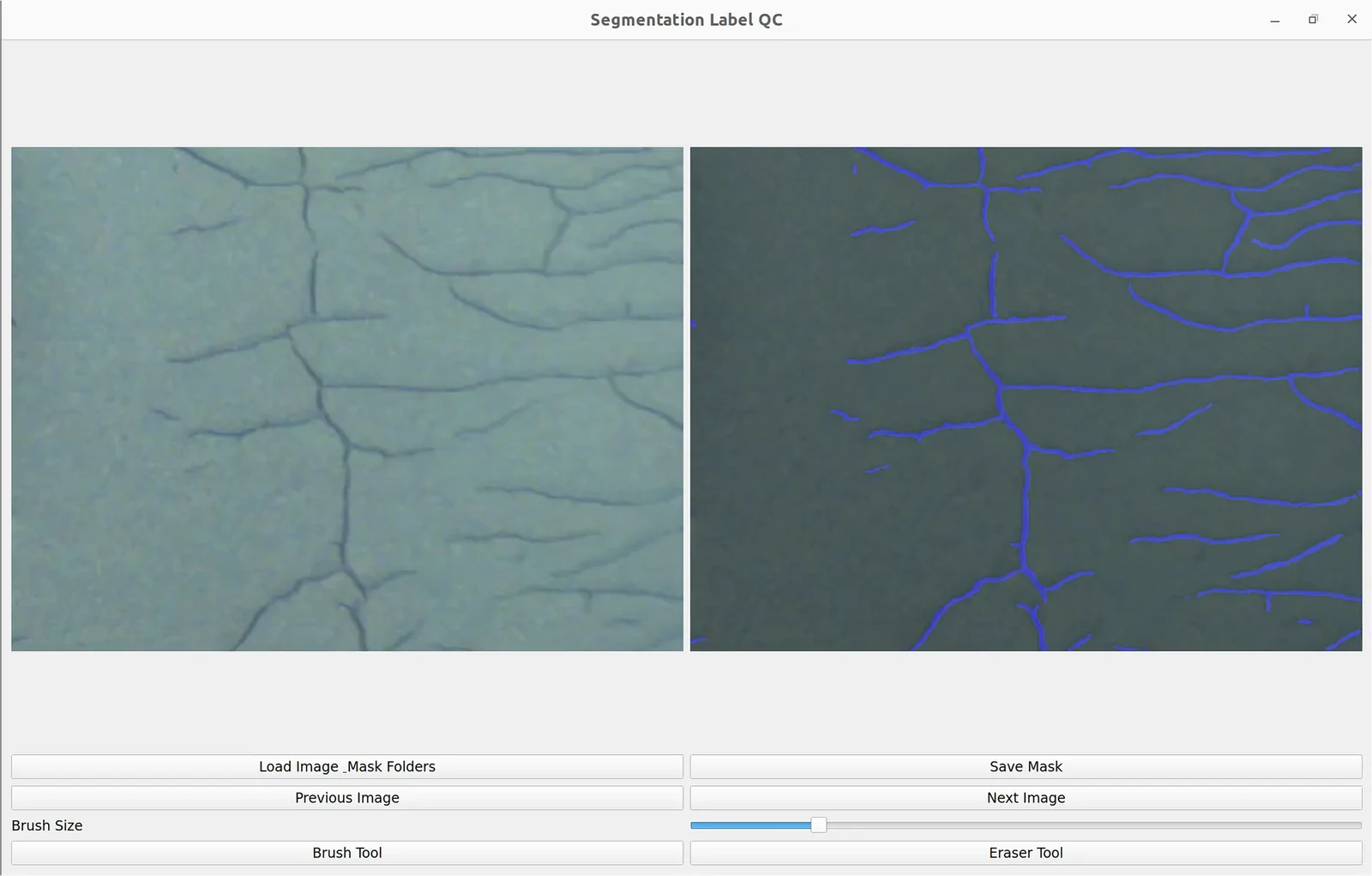
Snapshot of the QC tool interface used by annotators to manually review and correct pseudo-labels, addressing misclassifications and boundary errors in segmentation masks.

The generated pseudo-labels are then reviewed and manually corrected by experienced annotators using annotation quality control (QC) tools (see Fig. 4). This step aims to address any misclassifications or boundary errors in the segmentation masks. Manual corrections may include refining the edges, adjusting the size of detected defects, or correcting misidentified regions. This step is crucial for ensuring the accuracy of the annotations, especially where the automated models may have struggled. The annotators in this step are skilled in identifying coating defects, with expertise in electrode production and coatings.

#### Step 5: Iterative refinement and model retraining

Once the manual corrections are made, the corrected annotations are incorporated back into the dataset as ground truth labels. These refined annotations are then used to retrain the segmentation models. During retraining, an ensemble approach is employed where the outputs of all three models are combined to produce a more robust prediction. Each model in the ensemble is trained with different architectures or hyperparameters, helping to reduce bias and variance. The ensemble method benefits from the strengths of each model, leading to better overall performance and more accurate defect detection. After retraining, the updated segmentation models are used to generate new segmentation masks, which are again subjected to manual correction, forming a continuous loop of improvement.

Through this iterative process, the segmentation models gradually become more accurate and robust. With each iteration of manual refinement and model retraining, the models’ ability to detect and segment defects in electrode coatings improves, ensuring better accuracy over time. To minimize missed defects, we employed a multi-pass manual verification protocol involving domain experts to carefully review annotations. This rigorous process significantly reduces the likelihood of unannotated subtle defects.

#### Step 6: Final verification and model evaluation

Once the segmentation model has undergone sufficient iterations of training and refinement, the final set of annotations is reviewed by a team of expert annotators. This final verification ensures the accuracy and consistency of the ground truth data. The dataset is then divided into training and testing subsets for cross-validation and performance evaluation of the final segmentation model. Cross-validation ensures that the model is robust and can generalize well to unseen data, and performance evaluation metrics such as precision, recall, and F1-score are used to assess the model’s efficacy.

## Data Records

The CoatingVision dataset is available at Figshare^38^. It consists of 2,227 images, each with a resolution of 480 by 640 pixels and 3 color channels. It includes annotations for three main types of defects: Surface Crack, Delamination, and Pinhole. Among these, Surface Crack is the most prevalent defect, appearing in 1,947 images, which accounts for approximately 87.43% of the dataset. Delamination is relatively rare, with only 203 images (9.12%) showing this defect. Pinhole defects (see Fig. 6) are present in 519 images, representing about 23.30% of the total. Also, the dataset contains some unclassified defects, such as foreign materials and ambiguous irregularities, which are not explicitly categorized but may affect analysis. By mirroring the operational challenges faced in industrial coating inspections, CoatingVision serves as a highly relevant benchmark for developing and evaluating computer vision algorithms in manufacturing quality control. The dataset is structured in a way that supports a diverse range of image recognition tasks, including defect segmentation, defect detection, and multi-label classification.

## Defect segmentation

**Fig. 5:**
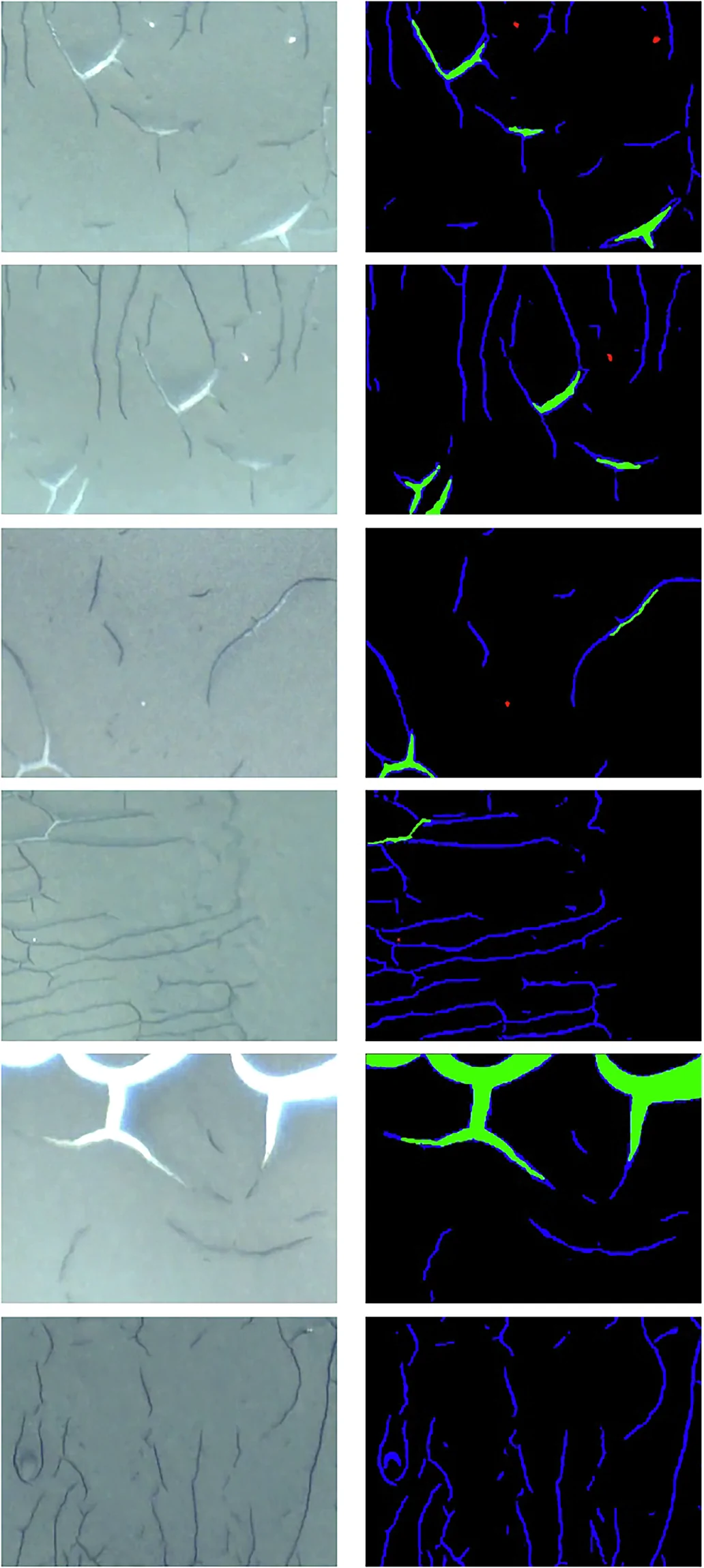
Sample images alongside their segmentation masks, with surface cracks highlighted in blue, delamination in green, and pinholes in red.

The segmentation masks in the CoatingVision dataset are provided in PNG format. Each image in the dataset is paired with a corresponding mask image that contains pixel-wise annotations for the various defects on the electrode surface (see Fig. 5). These masks are stored as multi-channel PNG images, where each defect type is encoded in a separate channel. Each segmentation mask is a 4-channel PNG image, where each channel represents a specific defect type. Each channel contains a binary mask where the value of each pixel indicates whether a specific defect is present at that location in the image. For example, in a 4-channel mask, the presence of a surface crack in a particular pixel is marked with a 1 in Channel 0, and similarly for delamination, pinholes, and unclassified defects in the corresponding channels. Each channel operates independently, enabling precise delineation of each defect type without overlap. The use of separate channels for each defect type ensures that multiple defects can be simultaneously identified at a given pixel location.

**Fig. 6:**
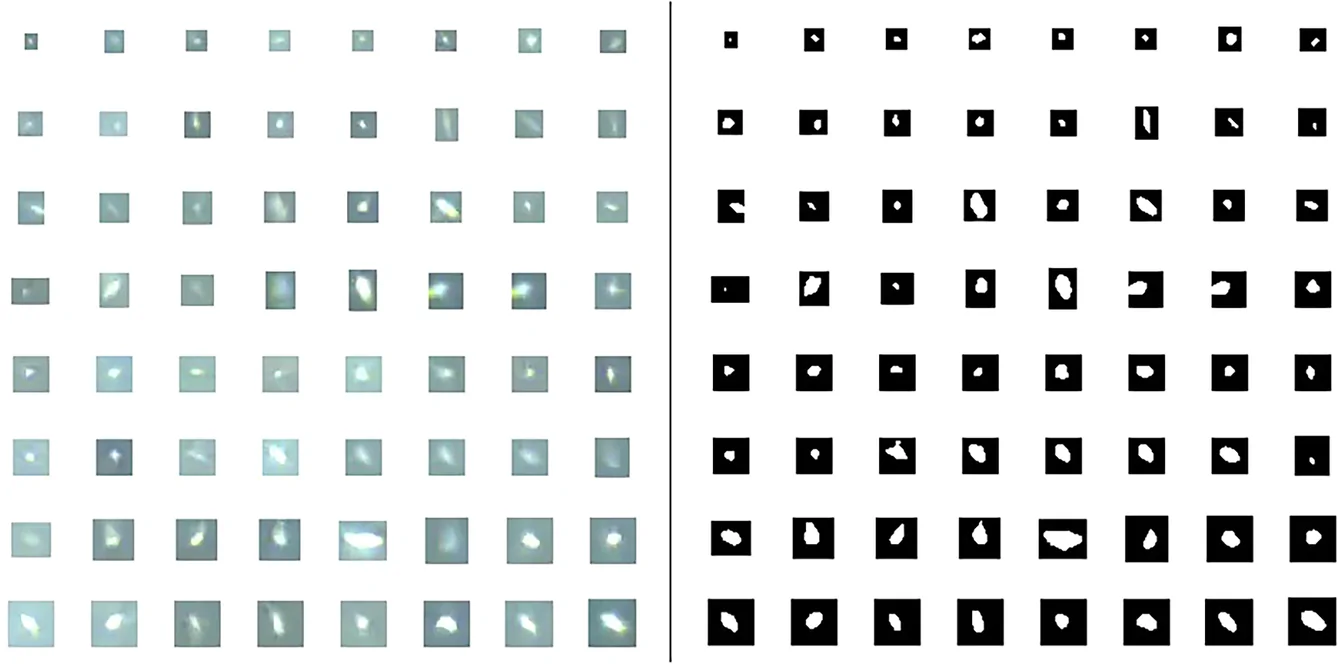
Visual overview of pinhole defects in the CoatingVision dataset and the associated segmentation masks.

**Fig. 7:**
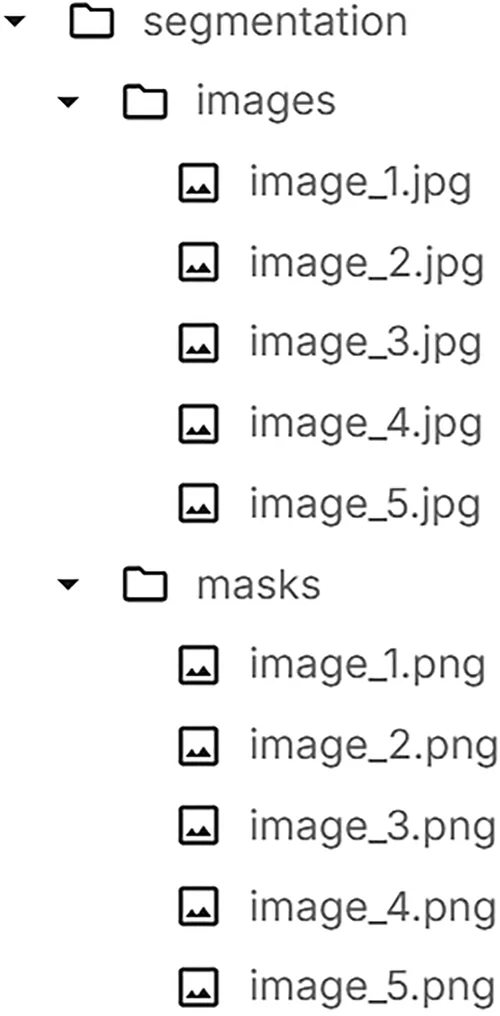
Directory structure of the CoatingVision segmentation dataset, with images and their corresponding masks stored in the ‘images’ and ‘masks’ folders.

The dataset is structured such that each image in the CoatingVision dataset is paired with a corresponding mask in the *masks/* directory (see Fig. 7). The images and masks are organized into the following subdirectories:

For each pinhole defect in the dataset, we measured the roundness to quantify how closely the shape of the defect resembles a perfect circle. The roundness is computed using the area and perimeter of the defect’s boundary, as defined in the following equation:

1 $${Roundness}=\frac{4\pi A}{{p}^{2}}$$

**Fig. 8:**
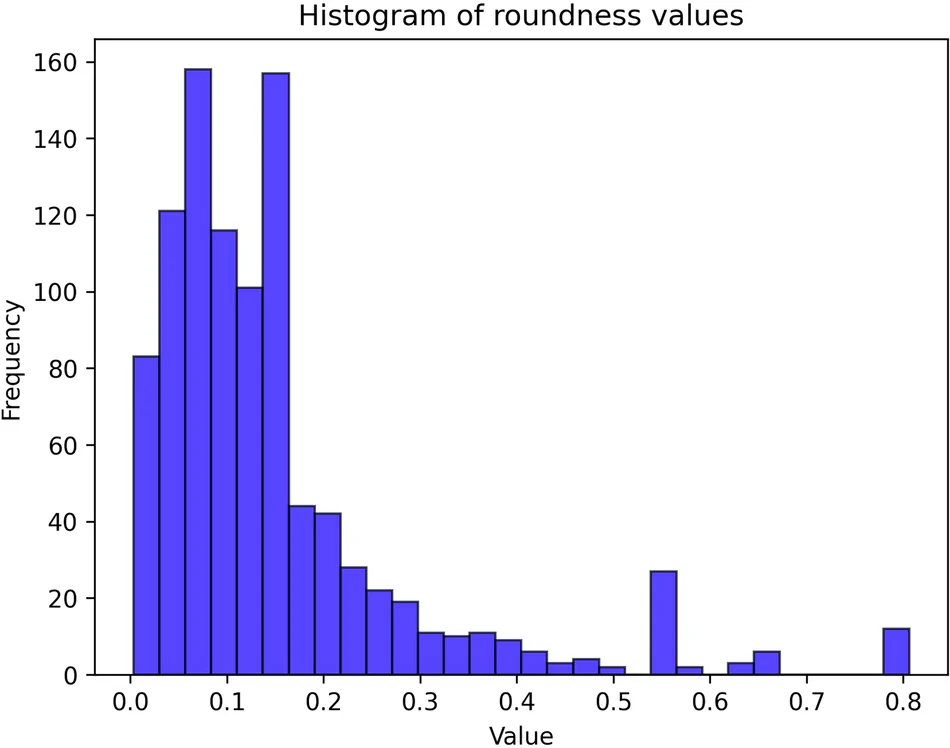
Histogram depicting the distribution of roundness values for all pinhole defects in the dataset.

Where, *A* is the area of the pinhole defect, which is the space enclosed by the defect’s boundary and *p* is the perimeter. To better understand the distribution of defect shapes in the dataset, we plotted a histogram of roundness values. The histogram (shown in Fig. 8) illustrates the frequency of different roundness values across all pinhole defects in the dataset. Most defects exhibit significant deviations from a roundness value of 1, indicating irregular shapes, while only a few are relatively circular.

The defects are defined using visual characteristics and established geometric criteria based on shape, scale, and depth. Surface cracks, delamination, and pinholes are distinguished by their unique geometric features. Surface cracks appear as linear fractures, delamination as coating separation, and pinholes as small voids.

### Defect classification

**Fig. 9:**
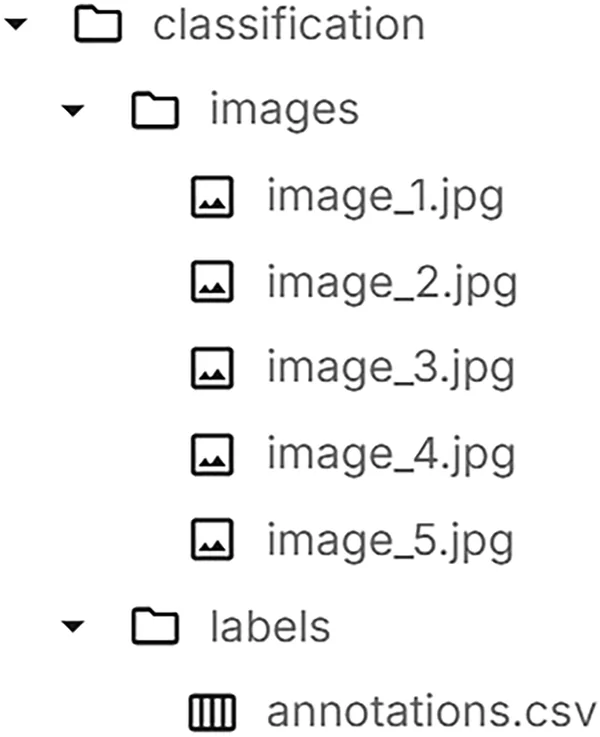
Directory structure of the Coating Vision classification dataset, with images in the ‘images’ folder and annotations in the ‘labels’ CSV file.

The CoatingVision dataset also includes labels for the multi-label classification task, where each image is annotated with one or more defect types. The defects are classified into four categories: surface cracks, delamination cracks, pinholes, and unclassified defects. The classification task requires determining the presence or absence of these defects in each image, with the possibility of multiple defects being present simultaneously. The labels for the multi-label classification task are provided in CSV format, where each row corresponds to a single image in the dataset (Fig. 9). Each image has a corresponding label indicating the presence or absence of each defect type.

### Pinhole detection

**Fig. 10:**
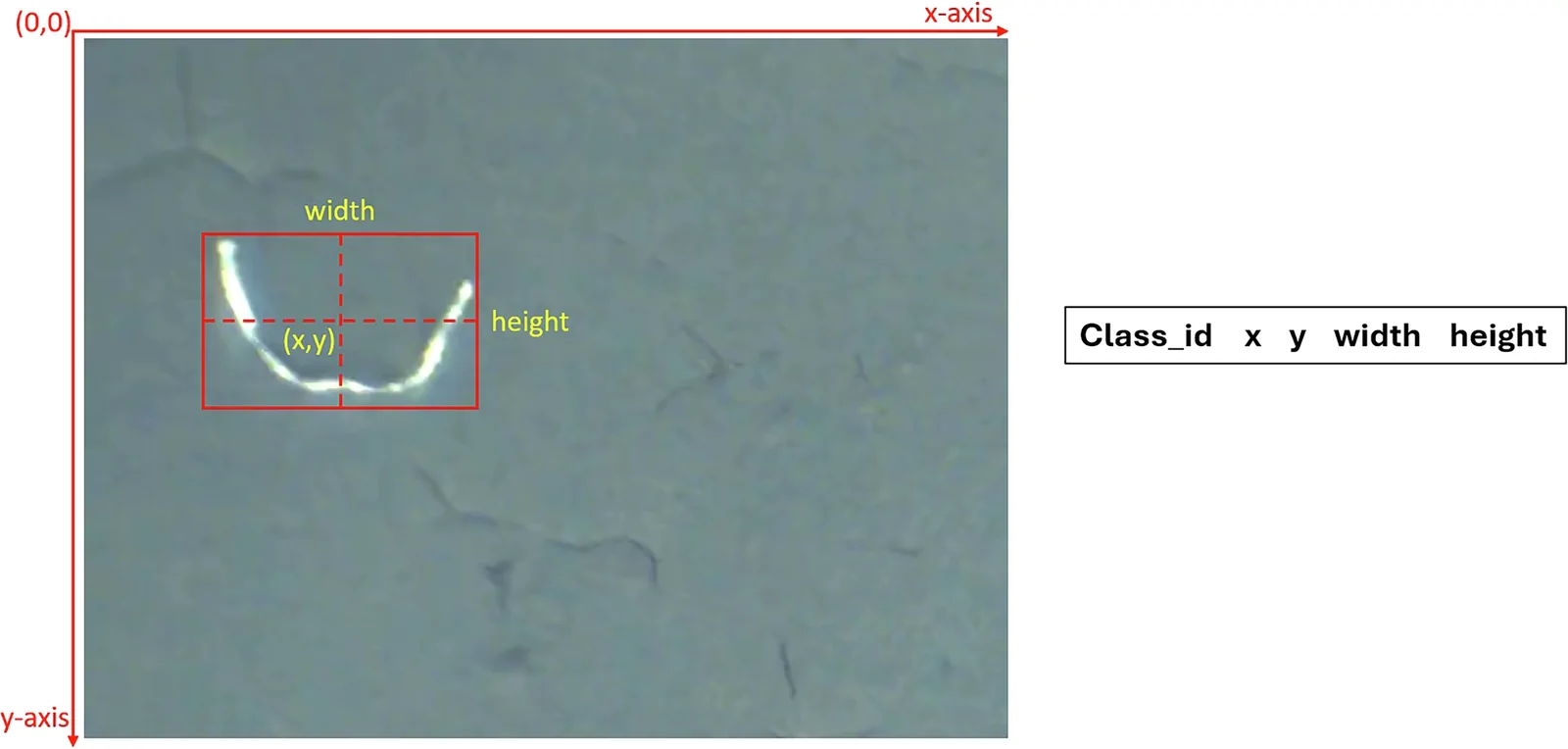
An example of a bounding box and YOLO annotation format.

Each image in the CoatingVision dataset is accompanied by a corresponding annotation file in YOLO text format (Fig. 10). The annotation files follow a specific structure, where each line corresponds to an object (i.e., defect) detected in the image. The format of the annotation file is as follows:

- **Class ID**: An integer indicating the defect type (e.g., 0 for pinholes).
- **X_center**: The normalized x-coordinate of the center of the bounding box, relative to the image width (a value between 0 and 1).
- **Y_center**: The normalized y-coordinate of the center of the bounding box, relative to the image height (a value between 0 and 1).
- **Width**: The normalized width of the bounding box, relative to the image width (a value between 0 and 1).
- **Height**: The normalized height of the bounding box, relative to the image height (a value between 0 and 1).

An example of an annotation file for an image with pinholes and unclassified defects is shown in Table 2.

**Table 2:** Example of an annotation file for an image containing pinholes and unclassified defects, with normalized coordinates (x, y) and dimensions (w, h) for bounding boxes.

Class_id	x	y	w	h
0	0.356	0.489	0.132	0.081
1	0.580	0.312	0.104	0.060

**Fig. 11:**
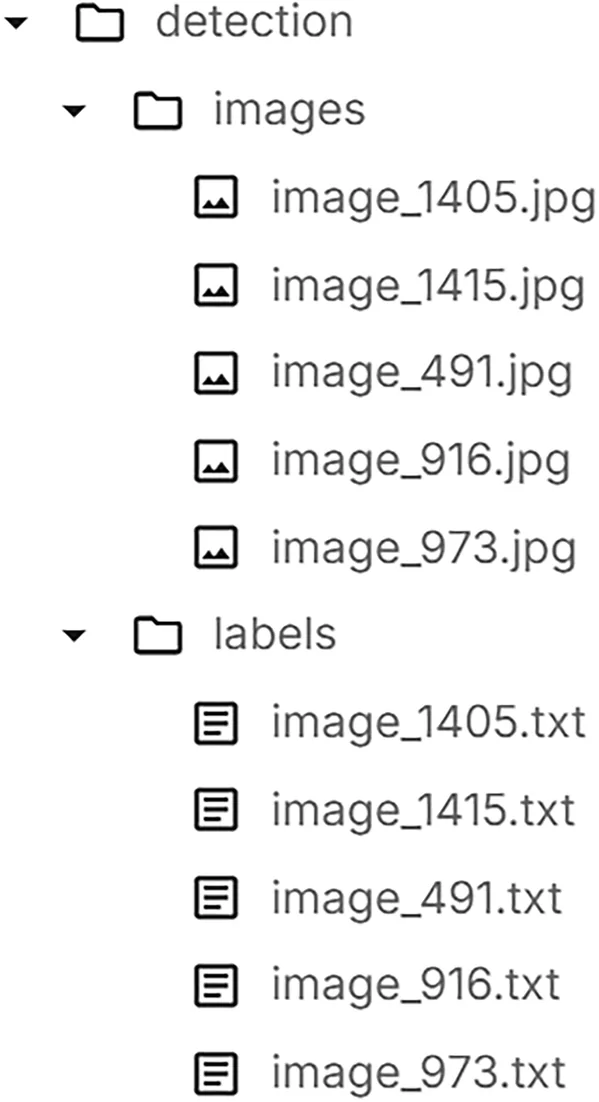
Directory structure of the CoatingVision detection dataset, with images stored in the ‘images’ folder and corresponding annotation.txt files in the ‘labels’ folder.

**Fig. 12:**
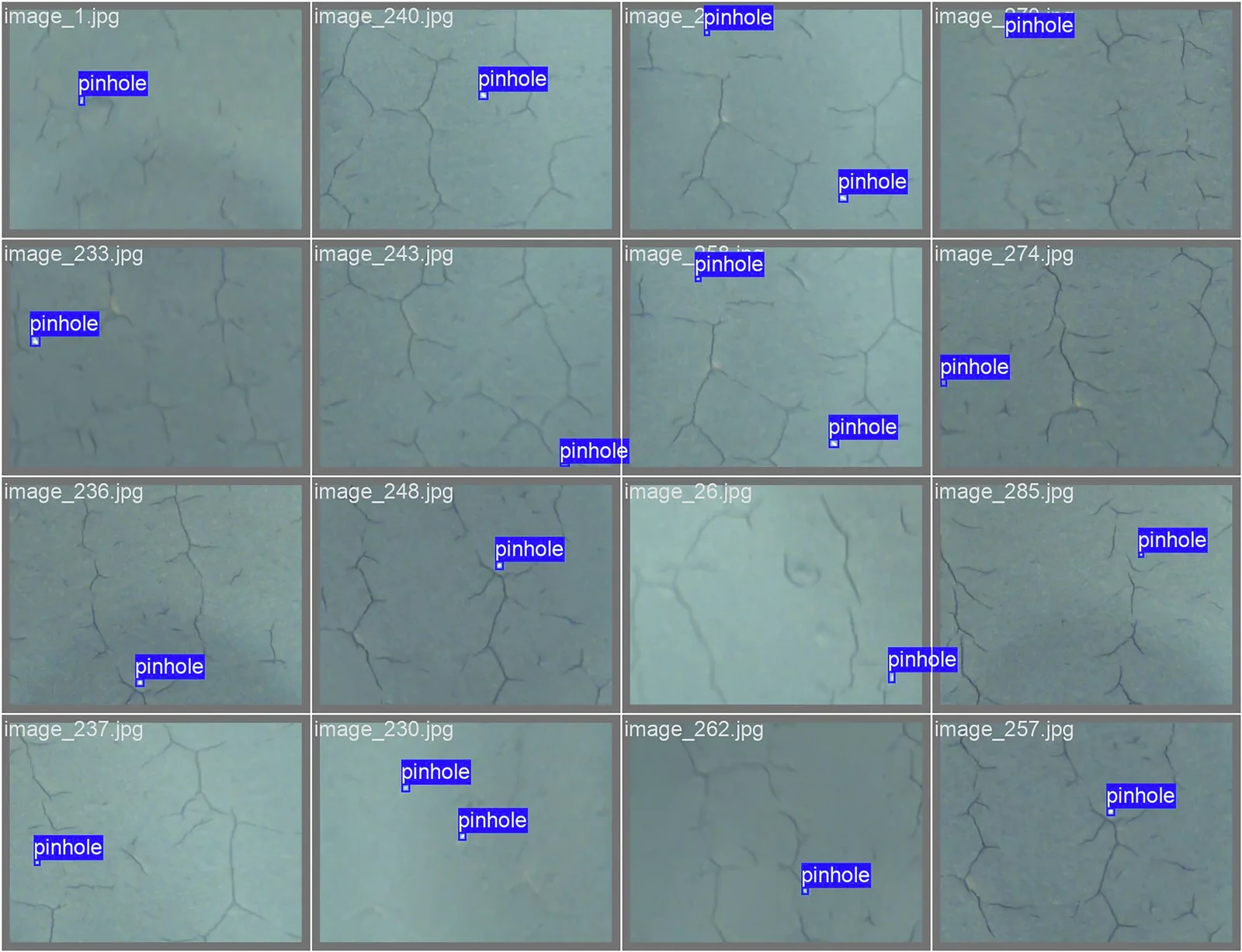
Sample images annotated with bounding boxes around pinhole defects.

This means that the first defect (class 0, pinhole) is located with its bounding box centered at coordinates (0.356, 0.489) and spans a width of 0.132 and height of 0.081, normalized to the image dimensions. The second defect (class 1, unclassified defect) is located similarly, with its own bounding box dimensions. Figures 11, 12 illustrate the directory structure of the CoatingVision detection dataset and examples of annotated pinhole defects, respectively.

## Technical Validation

To evaluate the technical effectiveness and statistical quality of the CoatingVision dataset, we conducted a series of supervised experiments on defect segmentation, object detection, and multi-label classification. These experiments aimed to establish benchmark performance metrics for future research and practical applications, while also validating the utility of the dataset across various defect detection tasks. The following sections detail the methods employed and the results obtained for each task.

### Defect segmentation

The segmentation task focuses on pixel-wise detection of defects on the electrode surface. We employed multiple distinct segmentation models for this task: a ResNet-based U-Net^39^, Feature Pyramid Networks (FPN)^40^, and LinkNet^41^. These models are well-established in the field of image segmentation and have demonstrated strong performance in similar defect detection applications^42–44^.

For the segmentation experiments, the dataset was divided into training and testing sets, with 80% of the images used for training and the remaining 20% reserved for testing. To assess baseline segmentation performance, two commonly used metrics were utilized: the Dice Similarity Coefficient (DSC) and Intersection over Union (IoU).

### Dice similarity coefficient (DSC)

The DSC is a widely used metric that quantifies the overlap between two sets. It is defined as:

2 $$DSC=\frac{2|A\cap B|}{|A|+|B|}$$

where *A* and *B* represent the predicted and ground truth segmentation masks, respectively, and |*A*| and |*B*| denote the number of pixels in each mask. The DSC ranges from 0 (no overlap) to 1 (perfect overlap).

### Intersection over union (IoU)

**Fig. 13:**
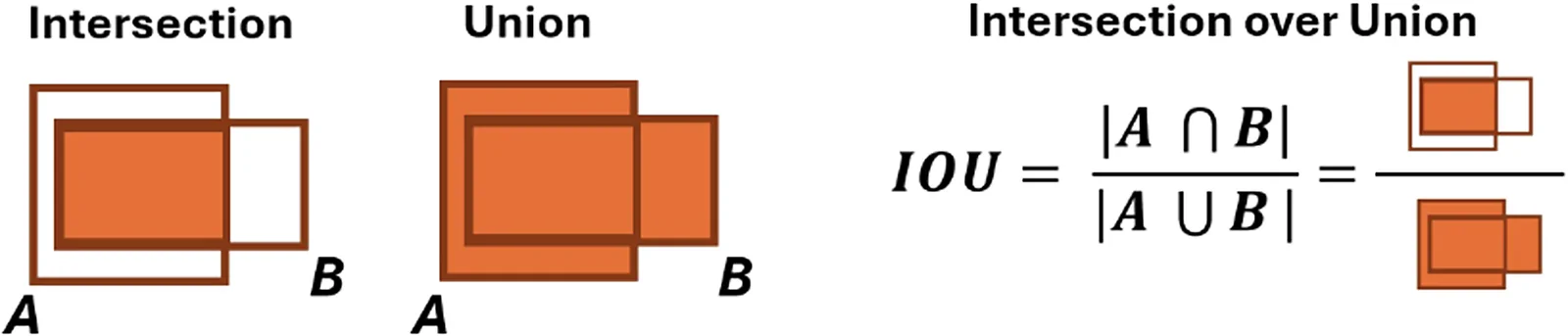
Schematic representation of IoU calculation.

The IOU is another widely used metric that measures the overlap between the predicted and ground truth segmentation masks (Fig. 13). It is defined as:

3 $$IOU=\frac{|A\cap B|}{|A\cup B|}$$

where |A∩B| is the number of pixels in the intersection of the predicted and ground truth masks, and |A∪B| is the number of pixels in their union. Similar to DSC, IoU ranges from 0 (no overlap) to 1 (perfect overlap), but it tends to penalize false positives more heavily than the DSC.

**Fig. 14:**
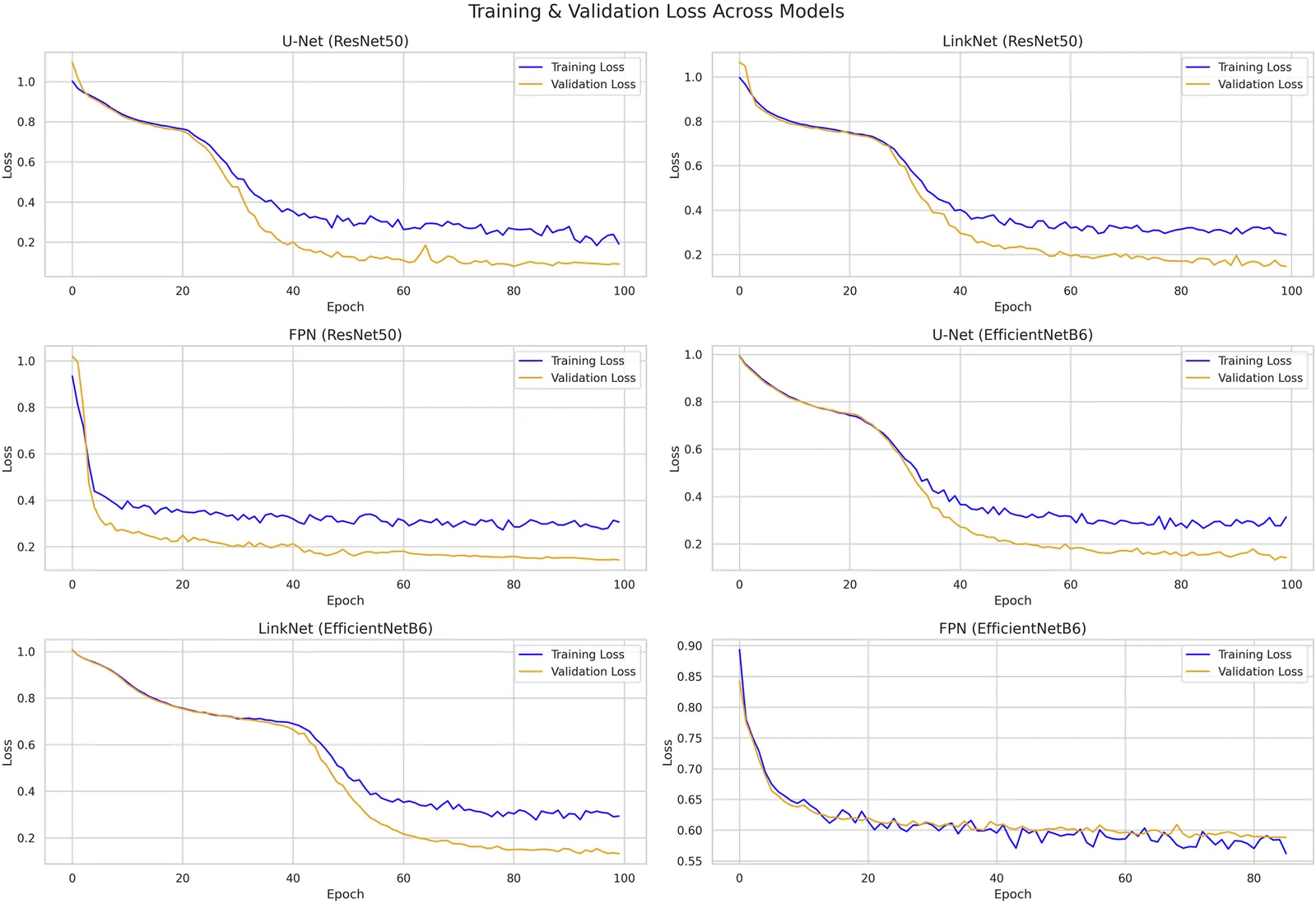
Training and validation loss across different models.

Both the DSC and IoU are essential for evaluating the quality and reliability of the CoatingVision dataset for defect segmentation tasks. These metrics assess the alignment between the predicted segmentation masks and the ground truth annotations, providing insights into the dataset’s accuracy in capturing defect characteristics. The supplementary table presents the evaluation results of the six segmentation model architectures, and Fig. 14 shows the training and validation loss across the different models.

### Defect detection

For object detection, we utilized the YOLOv11 (You Only Look Once) framework^45^, a widely adopted real-time object detection algorithm known for its speed and accuracy. The object detection task in the CoatingVision dataset aims to localize defects within the images using bounding boxes. The model was trained to predict bounding boxes around defects in the electrode surface, where each bounding box includes the defect location and its class label. To evaluate the performance of the YOLO model, we used the following commonly adopted object detection metrics: mean Average Precision (mAP), Precision, and Recall.

### Mean average precision (mAP)

mAP is the primary metric used for evaluating the overall performance of object detection models. It provides a summary of how well the model detects and localizes defects by calculating the average of average precision (AP) scores across different defect classes. The AP score is obtained by averaging precision values at various recall levels, considering different confidence thresholds for detection. The mAP value ranges from 0 to 1, with higher values indicating better overall performance of the model in terms of both localization and classification of defects.

It is defined as:

4 $${mAP}=\frac{1}{N}\mathop{\sum }\limits_{i=1}^{N}A{P}_{i}$$

Where N is the number of defect classes (e.g., different types of defects) and *AP*_*i*_ is the Average Precision for the *i*-th class. *AP* itself is computed as the area under the Precision-Recall curve for each class, reflecting the model’s precision at different levels of recall. A high mAP value indicates that the model has achieved both high precision and recall across all classes. Table 3 presents the evaluation results of the YOLOv11 and Faster-RCNN object detection models.

**Table 3:** Performance Metrics Object Detection Models.

Model	*mAP* @ 0.5	*mAP* @ [0.5:0.95]	Precision	Recall
YOLOV11	0.7956	0.5208	0.7588	0.7621
Faster-RCNN	0.7480	0.4420	0.7785	0.6705

**Fig. 15:**
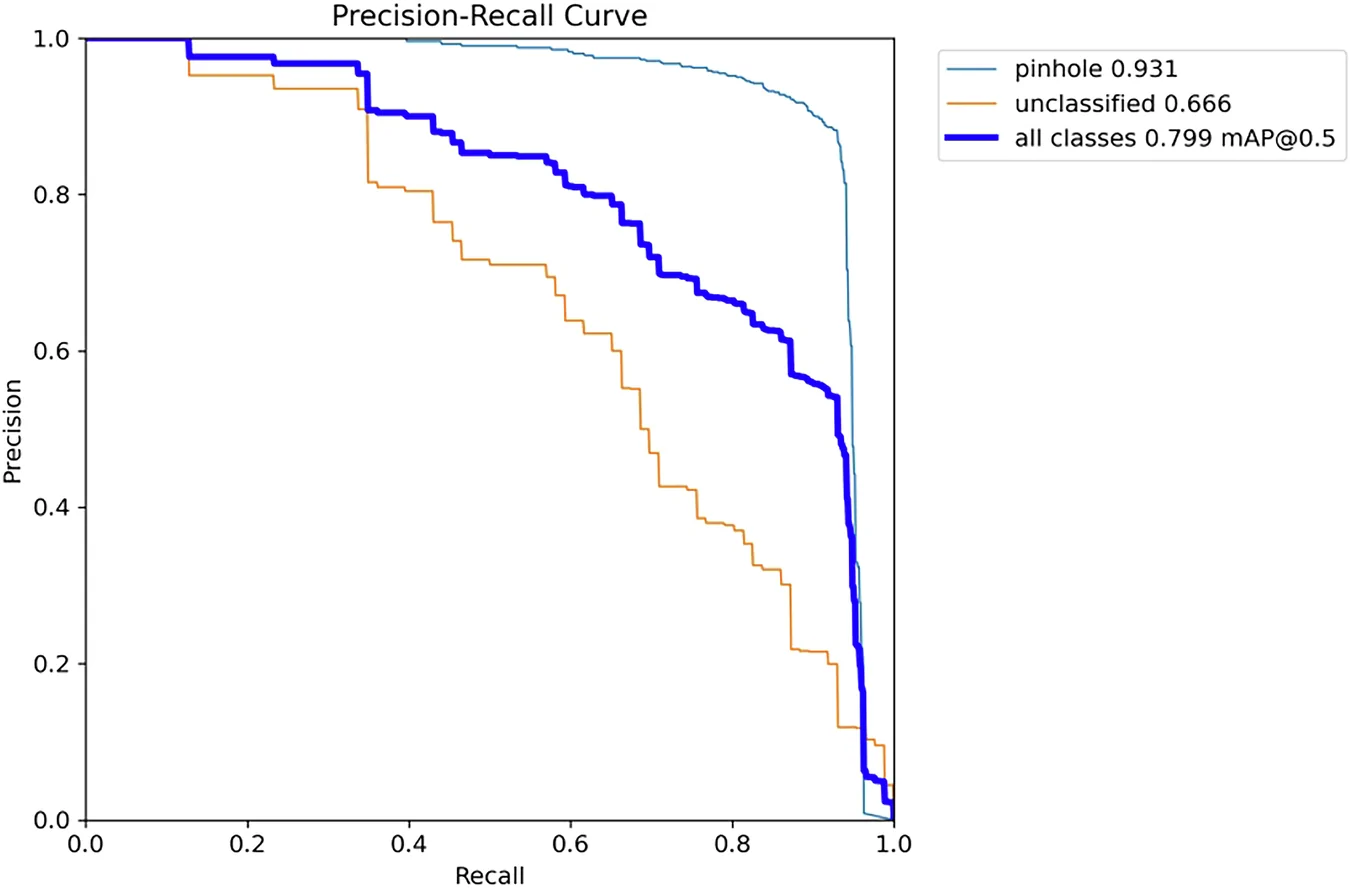
Precision-Recall Curve for YOLOV11 across defect Types.

Figure 15 illustrates the Precision-Recall curve for YOLOv11, showing the model’s ability to balance precision and recall across various defect types. The curve illustrates how well the model performs at different levels of confidence and recall, with the area under the curve (AUC) serving as a reflection of overall performance.

### Defect classification

The multi-label classification task involves predicting multiple defect types simultaneously for each image, where each defect class can be independently predicted as present or absent. This is particularly relevant in the context of the CoatingVision dataset, where a single image can contain multiple types of defects on the electrode surface. To address this task, we employed two well-known deep learning models: ResNet^46^, ConvNext^47^, and EfficientNet^48^. These models are recognized for their strong performance in classification tasks, leveraging advanced architectures that efficiently capture both local and global features within the images. The performance of the models was evaluated using two standard metrics: F1 Score Precision, Recall and Accuracy (Table 4).

**Table 4:** Comparative Performance Analysis of three Different classifications model Architectures.

Architecture	Surface Crack				Delamination				Pinholes			
	Precision	Recall	F1	Accuracy	Precision	Recall	F1	Accuracy	Precision	Recall	F1	Accuracy
ResNet^46^	0.9121	0.8736	0.8924	0.9074	0.7973	0.7266	0.7600	0.7963	0.7848	0.6902	0.7344	0.7768
ConvNext^47^	0.9374	0.8910	0.9135	0.9245	0.8740	0.6204	0.7257	0.7921	0.8684	0.6680	0.7551	0.8106
EfficientNet^48^	0.9383	0.8886	0.9127	0.9244	0.8777	0.6601	0.7535	0.8086	0.9229	0.7506	0.8276	0.8604

#### F1 Score

The F1 score is the harmonic mean of precision and recall, providing a single metric that balances both the precision (the ability to avoid false positives) and recall (the ability to detect all true positives). It is useful in multi-label classification, where multiple defect classes may be predicted for each image, and achieving a balance between precision and recall is critical.

The F1 score for a single label *i* is defined as:

5 $${F1}_{i}=\frac{2{.{precision}}_{i}\ast {{Recall}}_{i}}{{{precision}}_{i}+{{Recall}}_{i}}$$

The macro-averaged F1 score is computed by taking the average of the F1 scores across all classes:

6 $${F1}_{{macro}}=\frac{1}{N}\mathop{\sum }\limits_{i=1}^{i}{F1}_{i}$$

Where N is the total number of defect classes. A higher F1 score indicates a more balanced performance in terms of precision and recall for each defect class.

## Supplementary information

Supplementary Table

## Data Availability

The dataset is available at https://doi.org/10.6084/m9.figshare.29260121.v1.

## Code availability

The code for data annotation, model training, and evaluation is available on GitHub at https://github.com/vigsam-coder/CoatingVision. The dataset has been uploaded to Figshare and can be accessed at https://figshare.com/articles/dataset/29260121.
